# Hosting an Online World Café to Develop an Understanding of Digital Health Promoting Settings from a Citizen’s Perspective—Methodological Potentials and Challenges

**DOI:** 10.3390/ijerph19169969

**Published:** 2022-08-12

**Authors:** Joanna Albrecht, Anna Lea Stark, Eleana Dongas, Kamil J. Wrona, Christoph Dockweiler

**Affiliations:** 1 Department Digital Health Sciences and Biomedicine, School of Life Sciences, University of Siegen, 57076 Siegen, Germany; 2 Department Demography and Health, School of Public Health, Bielefeld University, 33615 Bielefeld, Germany; 3 Faculty of Health, University of Applied Sciences Bielefeld, 33619 Bielefeld, Germany; 4 Faculty of Engineering and Mathematics, University of Applied Sciences Bielefeld, 33619 Bielefeld, Germany

**Keywords:** digital setting, digital health, eHealth, health promotion, prevention, healthy settings, participation, citizen science, virtual world café

## Abstract

Brown and Isaacs’ World Café is a participatory research method to make connections to the ideas of others. During the SARS-CoV-2 pandemic and the corresponding contact restrictions, only digital hostings of World Cafés were possible. This article aims to present and reflect on the potentials and challenges of hosting online World Cafés and to derive recommendations for other researchers. Via Zoom and Conceptboard, three online World Cafés were conducted in August 2021. In the World Cafés, the main focus was on the increasing digitization in settings in the context of health promotion and prevention from the perspective of setting members of educational institutions, leisure clubs, and communities. Between 9 and 13 participants participated in three World Cafés. Hosting comprises the phases of design and preparation, realisation, and evaluation. Generally, hosting an online World Café is a suitable method for participatory engagement, but particular challenges have to be overcome. Overall café hosts must create an equal participation environment by ensuring the availability of digital devices and stable internet access. The event schedule must react flexibly to technical disruptions and varying participation numbers. Further, compensatory measures such as support in the form of technical training must be implemented before the event. Finally, due to the higher complexity of digitalisation, roles of participants and staff need to be distributed and coordinated.

## 1. Introduction

Digital health technologies have been shown to impact health promotion and prevention positively and can be used in various ways [1,2,3]. Thus, low-threshold accessibility of target groups, user-oriented technologies [4], and partly a higher reach, greater effectiveness, and time and cost savings of interventions are possible [5]. In addition, digital interventions can help increase digital competencies connected to health [6] and are also effective in improving health-related clinical outcomes [7].

Since 2015, when health insurance in Germany was obliged to provide health promotion and prevention services in settings stipulated in the prevention law, health technologies have opened new potential and a need for adaptation, especially for setting-related health promotion [8]. Digital innovations have been changing the conditions of our everyday life in various respects, for instance, in settings such as schools, companies, communities, or leisure clubs, and are thus social innovations. In the context of increasing digitalisation, the question arises as to whether new digital settings have emerged in recent years. According to the World Health Organization (WHO) [9] (p. 19), the term setting describes the place or social context in which people engage in daily activities in which environmental, organisational, and personal factors interact to affect health and wellbeing. Subsequently, settings refer to formal organisations regarding social systems, which can be distinguished from their environment by formal membership regulations and specific role and competence structures.

Settings have been changed in their structures and cultures as well as interactions and processes by digitalisation [10,11], entailing new implications for health such as the dynamic development of eSports or the manifestation of new health risks (e.g., through cyberbullying or the addictive effects of digital media) and health opportunities (e.g., through social cohesion or positive effects for personality development) through social media [12]. In addition, digital health promotion and prevention possibilities are emerging, and digital possibilities for participatory cooperation between the different target groups and actors in health promotion and prevention.

Therefore, the derivation of interventions for developing health-promoting structures digital settings, both at the behavioural and structural/environmental level, is of particular relevance. A fundamental prerequisite is developing politically and practically operationalisable definition of digital setting. This should go beyond existing definitions and consider the needs and requirements of setting members.

To do so, the project aimed to develop a citizen’s definition of digital and blended settings in the context of health promotion and prevention. Participatory-creative methods such as the World Café method by Brown and Isaacs [13] can be used. The World Café method was developed in the 1990s in the USA as a participatory approach to solving community-related problems as well as social issues, which enables collective knowledge-sharing between different key stakeholders and unites the perspectives of different stakeholders on problems and possibilities through lively exchanges of ideas and feelings [13]. So, this method is often used in health promotion and prevention research and practice to network different perspectives to uncover heterogeneous challenges, opportunities, and opinions [14,15,16,17].

Due to the dynamic development of the SARS-CoV-2 pandemic and the resulting contact restrictions, the analogue hosting of group-based research methods faced challenges and had to be realised in online formats. Various online World Cafés have been conducted for public health research [18,19,20] to connect (globally) and creatively discuss perspectives of different sized audiences in a comfortable atmosphere, for example, to facilitate and stimulate discussions between health educators to promote online learning [18], identify research priorities in mental health [19], or gain a better understanding of indigenous health issues [20]. In our research project, the hosting of participatory research was also necessary. Compared to previous research, three different online World Cafés were conducted, in which participants with different socio-demographic characteristics and diverse backgrounds in terms of target groups and settings were involved. However, the realisation and methodological discourse on the digital hosting of a World Café have been only marginally investigated so far [18,19,20]. So, beyond the content-related findings—which will be published in a separate article—insights linked to the methodological hosting of online World Cafés with members from different settings such as educational institutions, leisure clubs, and communities were preserved. To develop an understanding of digital health promoting settings from the perspective of citizens, the project research question was put: “How can citizens define the term ‘digital setting’ in the context of health promotion and prevention?”

From the context of online hosting in order to develop this content, this article is based on the following research question: “How can the research process be conducted in hosting online World Cafés?” So, this article aims to present and reflect on the design and hosting of the online World Cafés to derive methodological advantages, challenges, and recommendations for other researchers in hosting an online World Café.

## 2. Materials and Methods

To answer the project research question, three online World Cafés, each lasting approximately three hours, were hosted in August 2021 as an online format using the video platform Zoom and the digital pinboard. Conceptboard following the World Café method based on Brown and Isaacs [13]. As settings digitise and change differently and have different cultures, structures, and interactions [10,11], various understandings of a digital setting can be expected. Therefore, the three diverse settings “educational institution”, “community” and “leisure club” were selected for a closer focus of the study. Thus, each World Café focused on one of these settings, and the overarching question for each online World Café was adapted to the specific setting:

Online World Café 1: “What constitutes a digital educational institution?”

Online World Café 2: “What constitutes a digital community?”

Online World Café 3: “What constitutes a digital leisure club?”

Each of the three World Cafés developed a definition for a digital educational institution, a digital community, or a digital leisure club. The synthesis of these results led to an overall understanding of digital settings from the perspective of the setting members.

### 2.1. Underlying Idea 

**Figure A1:**
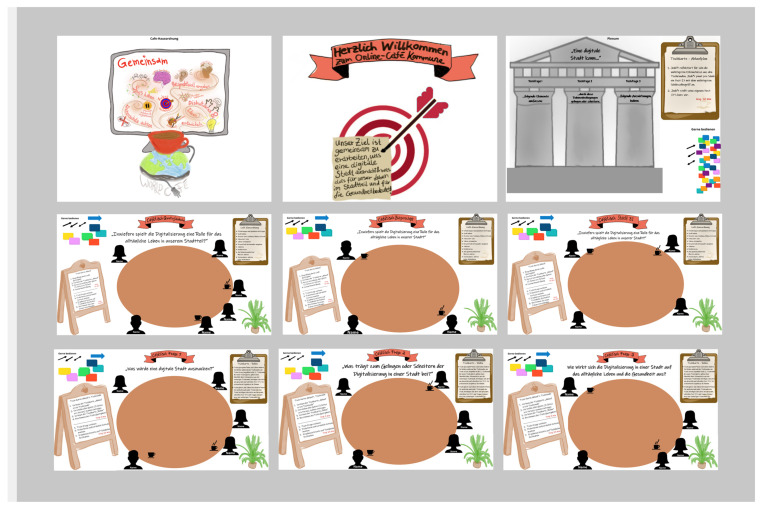
Empty digital pinboard before the online World Café “community”.

In the World Café, a larger group of participants (minimum 12) is enabled to hold conversations in small groups at different tables and develop their discussion dynamics. Participants then switch group tables in several rounds (each 20–30 min), which allows ideas to be passed on, perspectives to be linked, and collective knowledge of the large group to become visible. The results are recorded as notes or drawings on the tablecloths [13]. To realise this underlying idea in a digital format, nine tiles were created on a digital pinboard of the Conceptboard, which visualise the digital tables (cf. Appendix A Figure A1). This figure and all other figures on the digital pinboard are for visual illustration purposes only and do not represent content-related results. For this reason, the original German-language figures are shown.

**Figure 1:**
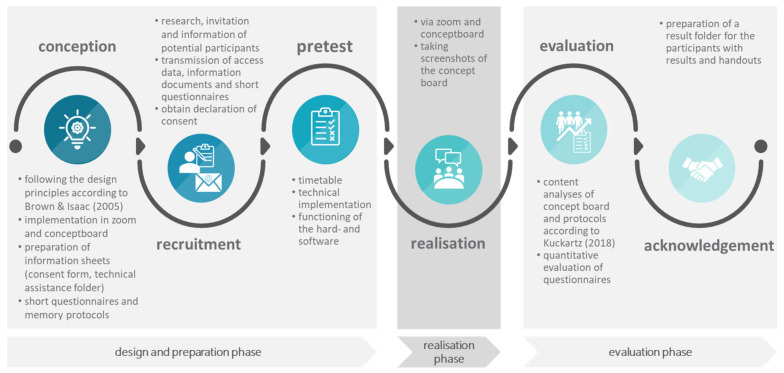
Process of hosting online World Cafés.

The course of the research process of the hosting of online World Cafés is shown in Figure 1. The design and preparation phase includes, on the one hand, the conception along the design principles according to Brown and Isaacs [13], the creation of survey instruments as well as the technical preparation in Zoom and Conceptboard. On the other hand, recruitment and pretest are part of the design and preparation phase. The subsequent realisation phase covers the actual implementation and the distribution of tasks among the various participants. The screenshots taken of the developed content (tablecloths at the digital pinboard) during the realisation phase are the object of evaluation in the subsequent evaluation phase. Once all the contents have been evaluated and written down, the participants receive an appropriate summary of the core findings in a results folder.

The methodological design of the online World Cafés followed the seven design principles and role allocation of the original (analogue) method by Brown and Isaacs [13]. The extent to which the online World Cafés were designed compared to the original method is explained below.

### 2.2. Phase 1: Design and Preparation Phase

The World Café tables were digitally illustrated via a digital pinboard. Participants were able to edit this digital pinboard and fill it with notes or other written and visual elements. To coordinate the planned discussions and the documentation of the results of the individual table rounds in small groups and the plenary, nine tiles were created on the digital pinboard (cf. Appendix A Figure A1). The top left tile shows the World Café etiquette, which provides the basis for joint work in the World Café. The tile in the top middle shows the overall goal of the World Café, namely, finding the definition of a digital educational institution, a digital community, and a digital leisure club, respectively. The tile at the top right visualises the task for the concluding plenary discussion, aiming at aiming at synthesizing the results. All other tiles visualise the tables of the table rounds. The tiles and their contents can be enlarged by the overall café host to allow all participants to be carried along on the board simultaneously. In order to guarantee a quiet atmosphere for the small group discussions, three Zoom breakout sessions were set up in each table round. The Zoom conference room was used for the plenary discussion.

*Roles*: When conducting a World Café, the scientists and participants take on different roles: An overall café host or hosting team, café guests, or an individual table host. The organisers of a World Café perform the role of the overall café host. They do not act as moderators or discussion leaders to reinforce their roles as café hosts rather than traditional presenters. They listen to the group discussions by mingling without disturbing participants. They introduce the principles and café etiquette to the participants during the event and strive to ensure that the participants feel comfortable and that they always keep “the big picture” of the event in mind. The role of café guests is taken by the participants invited to the event. In addition, at the beginning or end of the small group discussion in the first table round, the café guests choose an individual table host. The chosen table hosts of each table actively participate in the discussion without acting as moderators. They stay at the same table throughout the different table rounds and welcome the new guests after each table change. Further, they give an overview of the previous conversation to build a basis and facilitate the following discussion. In each table round, all table guests help to take notes and summarise important insights. Those new to a table bring in essential ideas from their previous round and make connections to the ideas of others [13].

In addition to the traditional roles of overall café host (OCH 1 and 2), individual table host (ITH 1, 2 and 3), and café guests, two additional roles were added for the realisation of online hosting: table observers (TO 1, 2 and 3) and technical assistant (TA). An overview of the people involved in the hosting team, their roles, and their functions are given in Table 1.

One scientist per table took over the role of the table observer during the discussion in the small groups (Zoom breakout session) and the plenary sessions. Instead of the café host who listens to each table, as in the original method, we decided to have table observers stay at each table. In the small group discussions, which took place via Zoom in the breakout rooms, the table observers only greeted the café guests at the tables and then passed the floor to the group. The table observer, therefore, did not participate in the substantive discussion and only intervened in the group discussions if there was a conflict between the participants if the discussion did not go further, if no one participated, or if the discussion was only about individual points of views and not about a common perspective. When the table observed had to intervene, the exemplary questions and phrases provided in the World Café method were used (e.g., What is still missing from the picture? What do we not see?). In addition, the table observers observed the discussions at their table and took notes to record the course of conversation in the memory protocol after the World Café. The technical assistant took over the technical and time management of the breakout sessions, served as a contact person in case of technical problems, and navigated the participants on the digital pinboard to the respective field via the sharing function of the Conceptboard. The different conception and deviation from the original method are shown below, along with the individual design principles.

(1) *Set the context*: This principle consists of explaining and defining the three key elements: purpose, participants, and parameters [13]. First, we tried to clarify the purpose of the World Café. The relevance of a participatory definition of digital settings from the perspective of setting members was stated. Thus, it is necessary to consider the perspectives of different settings members to depict needs and requirements heterogeneously and to develop a definition corresponding to the reality of the life of those affected. The small mixed groups are suitable for allowing everyone to have their say. Linking perspectives and knowledge is a suitable way to develop a definition. The choice of the event title “Online-Café-Knowledge creates the future-tracking down digital settings” is based on the purpose of the event. The expected outcomes are the collection of essential and relevant features of digital settings, with all participants adding to their knowledge and learning from other people or perspectives.

**Figure 2:**
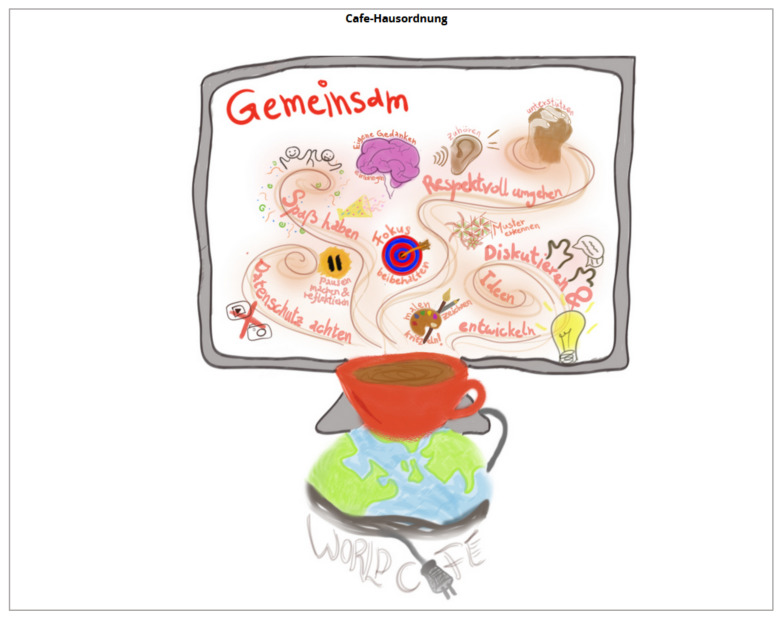
Conceptboard tile “etiquette”.

It is recommended that participants are briefed at the beginning of a World Café on the assumptions and etiquette of a World Café. This was visualised and made transparent in the digital pinboard via the café etiquette, according to Brown and Isaacs [13] (cf. Figure 2). The etiquette includes aspects such as “focusing on what is important”, “contributing one’s thinking and experience”, “listening to understand”, “connecting ideas”, and “looking together for patterns, insights, and deeper questions”. For the hosting of an online World Café, the aspect “respect data protection” was added to protect the personal rights of the participants. This ban is on image, sound, and video recordings [13].

When it became clear that the World Café could only take place digitally due to the SARS-CoV-2 pandemic, possibilities of digital hosting were considered. After selecting the Zoom and Conceptboard platforms, licences were obtained. Furthermore, it was ensured that each member of the hosting team had a digital device (computer or laptop), camera, and microphone. Information on the availability of the designated digital technologies and the planning of Zoom instruction and Zoom training for the participants of the online World Café was also planned as pre-event activities. As a post-event follow-up, the processing of the findings in the form of a summary of results was planned to offer the participants a benefit.

Furthermore, we determined the desired participants. The participants were selected to achieve a high degree of diversity. For this purpose, persons were recruited from different institutions in Germany, which can be divided into the three settings described above: educational institutions, communities, and leisure clubs. In the online World Café “educational institutions”, the secondary school, university, and adult education centre participated. Due to the online hosting and the reduced travel distances, it was possible to recruit nationwide. The online World Café “community” should be attended by various municipal institutions, e.g., the care counselling service, integration assistants, neighbourhood management, the digitisation office, and the science office. The online World Café “leisure clubs” should consist of a sports club, a senior citizens’ club, and a youth leisure club. Each World Café should involve at least five participants from each institution, so 15 participants are represented per online World Café. The members of an institution were selected from different status groups (e.g., service providers, heads of facilities, and members of a setting). Mixing the groups into small groups was aimed to guarantee the diversity of perspectives and a multi-faceted discussion. The heterogeneous distribution of characteristics was related to sex, age, and professional status (within the institution).

Recruitment was carried out via invitations by e-mail by contacting the heads of the facilities as multipliers on the one hand and the facility members directly on the other. The heads of facilities were informed about the research project and asked to invite people from their facility’s various previously defined status groups to participate in the World Café if they were interested. In addition, the contacted heads of facilities and setting members received a flyer with information about the research project, the World Café, the data protection regulations, and the further procedure. Interested persons were instructed to register for the World Café by e-mail or telephone. The project staff then selected participants. Due to a lack of response and many cancellations (due to holidays, illness, or lack of time), recruitment was expanded. Thus, other adults and senior education institutions were contacted in addition to a specific type of adult education centre (“Volkshochschule”). Recruitment from secondary schools was expanded to primary and vocational schools and private tutoring institutes. Within the community, more individual citizens from the whole city were recruited rather than focusing on a specific district. After signing the consent form, participants received a schedule for the online World Café and the access link for the Zoom meeting, preparing them for the event.

**Figure 3:**
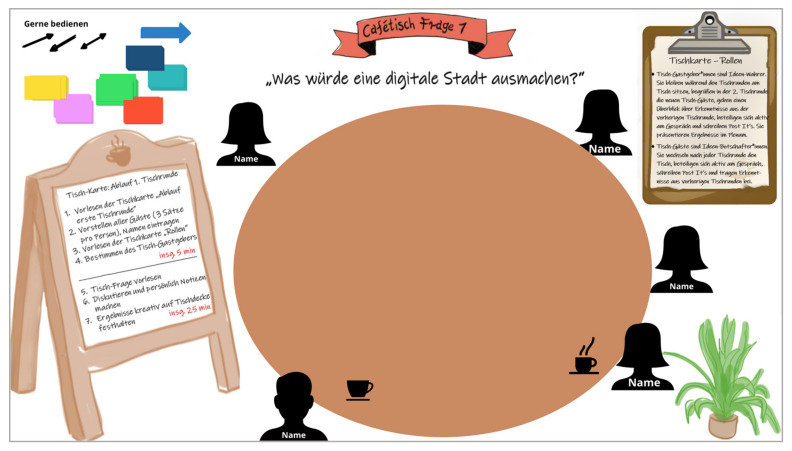
Conceptboard tile “café table question 1”.

(2) *Create a hospitable space:* This creates a welcoming, trusting environment where everyone feels comfortable and promotes mutual respect [13]. Those creations already took place with the design of personal invitations for the participants. To create an inviting, trusting environment in the virtual setting as well, design elements associated with an analogue café were used for the design of the information materials, welcoming presentation, and the digital pinboard, for example, original drawings, licence-free pictures of coffee dishes, menu boards and cards, tables, and plants (cf. Figure 3). Lounge music was also played during table changes to bridge pauses and waiting times.

During the World Café, the staff wore informal clothing. Name tags were displayed via Zoom. The intended communication at eye level was facilitated by Zoom, as all participants took the same position through the Zoom tile view. Nonetheless, the scientists’ provision of coffee and snacks was impossible due to the participants’ distance and the associated logistical challenges.

(3) *Explore questions that matter*: This concept aims to draw participants’ attention to issues that matter to them and promote community engagement [13]. Therefore, questions that matter is defining features of the World Café approach. According to Brown and Isaacs [13], the recommendations for question development were considered in the table questions’ design by formulating open questions that are suggestion-free, encourage creativity, and aim to generate a shared perspective [13]. To answer the overarching question, a total of four table questions per World Café (at four tables: one within the members of an institution, three mixed groups independent of the institution) were designed. Deviating from the method, the three World Cafés did not discuss exactly the same questions. The overarching question and the table questions were adapted to the focused setting in the World Café. This deviation can be justified by the heterogeneity of settings (e.g., different structures, cultures, interactions, and processes), which suggest a differentiated understanding of a digital setting. 

Another modification compared to the original method was made due to the heterogeneity of the participants. To first create a shared perspective on the topic of digitalisation in their institution, an institution-internal small group discussion was held. Subsequently, three small group discussions with mixed table groups were to take place to achieve networking of ideas in the sense of the World Café method. In the three table questions, relevant components of the definition of digital setting were to be addressed individually so that an overall picture of the definition could emerge in the plenary. Relevant components of the definition were defined as elements of a digital setting, factors for the success or failure of a digital setting, and the effects of a digital setting on health and living together.

Examples of questions and tasks in the online World Café “educational institution” are shown in Table 2. All developed questions were tested for comprehensibility and answerability in a pretest with setting members.

**Figure 4:**
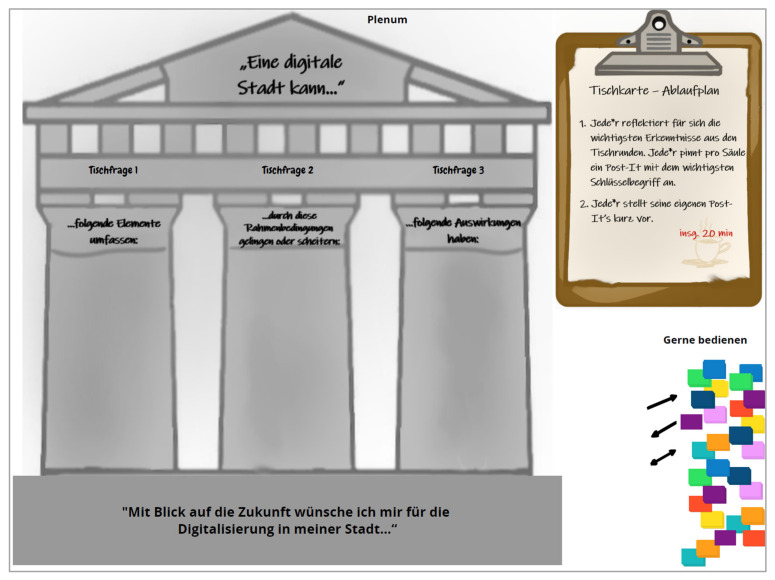
Conceptboard tile “definition house”.

To answer the overarching question in the plenary work, a “definition house” was developed and visualised (cf. Figure 4), containing the relevant results from the previous table rounds and the relevant components of the definition in the form of three columns. Here, the sorting and prioritisation of the table contents should take place.

The first task in the plenum was for each person to reflect on the essential findings from the table rounds. Each person pinned a Post-it with the essential key term per column. The second and third tasks were carried out for each of the three columns. Therefore, each person briefly presented their Post-its. The responsible table host led the procedure by collecting the Post-its. In the next step, the essential Post-its were prioritised using the number cards 1–3. As a final task in the plenary, the participants were asked to answer from the perspective of their role (status group), and which aspects are vital for them concerning the future in their setting.

(4) *Encourage everyone’s contribution*: To create the interrelation between the “I” and the “we”, each individual is encouraged to contribute and get involved [13]. Various actions were planned to encourage all participants to contribute and participate per the design principle. To create a low inhibition threshold for active participation, the online World Cafés were not recorded in auditory or visual form. The invitation already pointed out the requirements for successful participation, e.g., the availability of good internet quality, a digital end device, and a microphone and camera. Instructions for Zoom were also provided. 

In addition, to ensure that participants can use the functions of the Conceptboard independently, a Conceptboard exercise was integrated into the first small group discussion. To inspire creativity and participation, the café etiquette included the following phases: “Play! Doodle! Draw!” and “Contribute your thinking and experience”. The table observers reminded the café guests of these principles. There was enough space on the digital tablecloth to be creative. Likewise, it was made clear that verbally and on the Conceptboard that there are no wrong answers and that all contributions have merit.

(5) *Cross-pollinate and connect diverse perspectives*: It is essential to use the creative potential of living systems by purposefully increasing the diversity and interconnectedness of different perspectives while keeping the specific focus on the essential questions [13]. According to this plan, the breakout sessions were prepared in the Zoom conference room. To achieve the networking of different perspectives, in deviation from the original method, no random mixing of the groups was aimed for, but a targeted mixing of the participants in the small group discussions was planned. The participants were designated as table hosts and so-called idea keepers and were also addressed as such verbally. The task here was to work out shared insights and not to filter out differences. For this purpose, the registered participants were noted according to institution and status group. The table groups independent of the institution were composed as heterogeneously as possible regarding status groups and institutions. 

(6) *Listen together for patterns, insights, and deeper questions*: According to Brown and Isaacs [13], collective thinking is based on mutual listening, and sharing. To anchor this design principle in the online World Café, the World Café etiquette was creatively presented on a separate Conceptboard tile and introduced in the plenary (cf. Figure 2). Likewise, the central question of the World Café was visualised to draw attention to the bigger picture continuously. All participants could therefore access the World Café etiquette at any time. Furthermore, the intended collective thinking was strengthened by the wording of the questions, which included the phrase “from our common perspective”.

(7) *Harvest and share collective discoveries*: Finally, it is essential to make the jointly acquired knowledge visible in an action-oriented way [13]. Following the principle “None of us alone knows as much as all of us together”, the online realisation of the World Café tried to offer as many different possibilities as possible for the creative documentation of the discussions. Different coloured Post-its and various arrows were made available via the Conceptboard. In addition, design functions for the Post-its such as “reduce/enlarge font size”, “change font colour” and “change font” were introduced in the Conceptboard exercise.

Compared to an analogue World Café, the digital documentation of results also changes the data basis used for evaluation. As a basis for the evaluation, screenshots of the edited digital pinboard were made. As in the analogue hosting, the digitally labelled tablecloths served as central results in the online World Café. Each table observer created memory protocols after each event to collect main contents that were not recorded on the digital pinboard or particularities in the discussion dynamics or the course of the event. Key statements and features were recorded in these. A memory protocol was particularly suitable because the table observer could concentrate entirely on conducting the conversation and observing the situation during the events. Additional contextual information can be recorded compared to an audio recording [21].

In addition to the conception of the online World Café, a short questionnaire was developed to check and describe the heterogeneity of the participants. Focused characteristics were socio-demographic data such as age, sex, status group/professional position within the institution, focused setting, and affinity for technology. For better comparability, status groups were grouped as follows: (1) (department) heads or coordinators, (2) course or programme directors, (3) administrative staff, and (4) course or programme participants. Technology affinity is understood as a personality trait of the participant that is expressed in a person’s positive attitude, enthusiasm, and confidence towards technology [22]. Accordingly, it maps the technical interest and acceptance of technology and positively influences the knowledge about and experience with technology. The questionnaire for recording attitudes to and use of electronic devices (TA-EG questionnaire), according to Karrer et al. [22], was used to determine the affinity. On a total of four scales (enthusiasm for technology, competence in dealing with technology, the positive impact of technology, and negative consequences), the participants rate themselves on a five-point Likert scale for each statement [22]. The entire short questionnaire was checked for comprehensibility and functionality in a pretest and was slightly adapted.

## 3. Results

Based on the further phases outlined in Figure 1, the hosting of the online World Café is explained in more detail. 

### 3.1. Phase 2: Realisation

The online World Cafés was conducted on different afternoons in August 2021. After the first online World Café was hosted, two significant methodical changes were made. Firstly, the question formulations at the tables were adjusted. 

Thus, the online World Café on the setting “educational institution” showed that the wording “from our common perspective” makes it difficult to answer the questions because the individuality of the participating persons and institutions is downgraded, which the participants found obstructive. Secondly, it became clear that the time slots chosen for the individual table rounds were insufficient for an adequate discussion. Therefore, the schedule of the second and third World Cafés was adjusted by deleting one table round and reducing the plenary task. Consequently, only three table rounds were held (one within the institution and two across institutions). There was a focus on definition building in the definition house. The prioritisation and sorting of key terms were henceforth done by pinning the Post-its, which was more efficient overall.

For a better understanding, the exact timetable of the online World Cafés is described in detail below.

*Arrival and welcoming (2:30–2:50 p.m.)*: The participants were initially admitted from the Zoom waiting room to the Zoom room. They were welcomed and received an organisational briefing (data protection rules, technical information, advice on technical support in case of problems) and thematic (short project introduction, event objective, and process). The role allocation (cf. Table 1) was introduced. The participants were also introduced to the house rules of the café (café etiquette). A PowerPoint presentation via the screen sharing function of Zoom was used for the introduction. Then the café guests received an introduction to the Conceptboard tool. For this, the PowerPoint presentation was ended, and the Conceptboard application was made visible via the screen-sharing function of Zoom. The access to the digital pinboard and the primary navigation was briefly explained in the process. This was followed by the announcement of the first table round (internal to the institution) and the transition to the breakout rooms of Zoom. Throughout the facilitation, important information (such as phone number from technical support, privacy notice, and link to the Conceptboard) was written in the Zoom chat. In addition, breakout sessions were set up according to prior planning. 

*Discussion in small groups (within the institution) (2:50–3:20 p.m.)*: Within the first breakout sessions, the first small group discussion took place at the “digital tables”. Participants from the same institution formed small groups to facilitate access to the research topic and the first discussions. The table observers welcomed the small groups and received a detailed introduction to the functions of Conceptboard. After all café guests in the small group had access to the digital pinboard, a small exercise was conducted by having all café guests type their names and draw a Post-it on the table. After the exercise, the table observer announced that he or she was now withdrawing from the overall café host role, merely observing and handing over the floor to the small group. In doing so, he pointed out the schedule shown, which the small group read. This schedule includes the introduction of all participants, the reading aloud, discussing and answering of the table question, as well as the determination of a table host as the keeper of ideas. Afterward, the discussions took place in small groups, and the results were recorded on the “digital tablecloths”. A timer was set on the breakout rooms to show the remaining time.

*Presenting the results in the plenum (3:20–3:30 p.m.)*: After the first table round, the breakout sessions ended, and the café guests returned to the Zoom plenary. Then the designated table hosts presented the results of their small groups in the plenary. For all café guests to view the correct “digital tablecloth” during the presentation, the Conceptboard function “public viewing” was activated by one of the overall café hosts. This function allows zooming and navigating on the Conceptboard for all guests on the digital pinboard. During the presentation of the results, the technical assistant prepared new breakout sessions for the next round of tables. The participants from different institutions and status groups were as heterogeneous as possible. After the presentation of the results, the next cross-institutional table round was moderated, and the prepared breakout sessions were started. After the breakout sessions had started, the technical assistant made a screenshot of the previous results on the digital pinboard to save the intermediate step.

*First round of discussion in mixed groups (3:30–4:00 p.m.)*: The new, mixed small groups now discussed at a total of three digital tables on the digital pinboard, each working on one of the three given table questions. Again, a schedule was visualised. The tasks were: introduce all guests, read out the table card “Roles”, determine the table host, read out the table question, discuss and make personal notes, and creatively record results on the “digital table cloth”. The table card “Roles” contains a short description of the roles of “table host” and “café guests”. After determining the table host per table, the table question was discussed, and the discussion was visualised on the “digital tablecloths”. The technical assistant was informed which person was designated as a table host. This was important so that the table hosts were not divided into new groups when the following breakout sessions were created. When the time was up, the breakout sessions ended, and the café guests were brought back to the Zoom plenary. 

*Coffee and exercise break (4:00–4:15 p.m.)*: After the café guests returned to the Zoom plenary, the coffee and exercise break of 15 min was announced. The break served as a screen rest for the café guests. For orientation purposes, the PowerPoint presentation slide was shown to the participants, indicating the time the event was to be continued. Lounge music was also played. In addition, the technical assistant took screenshots of the digital pinboard. The last breakout sessions were set up during the break, aiming at a new heterogeneous mix of small groups. The selected table hosts stay at their “digital tables” during the second table round, while the other participants get together at another “digital table” in newly mixed groups. 

*Second round of discussion in mixed groups (4:15–4:45 p.m.)*: This time, the second and last mixed table round were moderated, and the breakout sessions were started. The schedule for the second table round was also visually depicted: The table host welcomes the café guests, all guests introduce themselves, the table question is read out, the table host gives an overview of the conversation so far, the discussion takes place, and personal notes are taken, all guests contribute insights from the previous rounds, results are creatively recorded on the digital tablecloth. 

*Presentation of results in the plenary (4:45–5:00 p.m.)*: After the small group discussions at the digital tables, all participants returned to the plenary, where the event’s overall goal was pointed out again. This was followed by the presentation of the “digital tablecloths” by the respective table hosts. The technical assistant also took a screenshot after this presentation of the results.

*Final discussion and plenary work (5:00–5:25 p.m.):* After the presentation of the results, the final discussion and task in the plenary was moderated. The tile “definition house” was used for this. A schedule was also visualized here, including the reflection, and writing down of the essential findings from the table rounds. 

*Acknowledgements and farewells (5:25–5:30 p.m.)*: Finally, there were farewells by the overall café hosts and an acknowledgement of participation. To release the participants from the event with a positive, creative impulse, they were finally asked to leave a last term or sentence on the digital pinboard, which should contain a personal wish for the future from the perspective of their role (status group). In the concluding words, the café guests also received a note that a summary of results was to be sent to them after the evaluation was completed. The hosting team remained in the Zoom channel until all café guests had left the Zoom room. After the event, a final screenshot was taken from the digital pinboard, and the table observers wrote their memory protocols.

### 3.2. Phase 3: Evaluation

The extent to which the online World Cafés were evaluated is explained below. To make it easier to conclude, the characteristics of the participants are first described.

Characteristics of the participants: The group size for all online World Cafés was below the desired group size of at least 15 participants and, in one case, below the minimum size of 12, according to Brown and Isaacs [13]. The characteristics of the participants in the online World Café are shown in Table 3. Thirteen people from various educational institutions participated in the online World Café “Educational Institution”.

Of the original 16 registrations, two cancellations were at short notice due to illness and other appointments, and one was a no-show. For the “school” setting, two persons from a secondary school and one from a primary school participated. The setting “adult education” was composed of three members of an institution for senior citizens’ education and two members of an adult education centre. A total of five persons were members of a university. In total, five persons were male, and eight were female. The age range was between 23 and 72 years. Some of the participants assigned themselves to several status groups simultaneously. There werre eight students, three teachers, and three coordinators.

The online World Café “community” took place with twelve participants. One person did not show up for the event despite having agreed to attend. For the “neighbourhood management” setting, four people from the neighbourhood work in one district took part, as well as one employee from a separate neighbourhood café. Three citizens formed the “citizenry” of the selected city. From the same city, four municipal employees participated as “employees” of the city. Among the participants, four are male, and eight are female, with an age range from 28 to 67 years. Considering the multiple selections, six participants assigned themselves to the status group citizens, four to the status group managers/coordinators, and two to the status group administrative employees.

Nine people took part in the online World Café “leisure club”. There were three last–minute cancellations, partly due to health reasons, and one participant joined the event a little later. Of the participants, three are male, and six are female with an age range of 21 to 68 years. Four people belong to the status group “course leader”, another four to “course/programme participant”, two to “institutional leader or coordinator” and another two to “administrative staff”, with some people belonging to several status groups at the same time.

Technology affinity: The TA was evaluated per participant by calculating the scale means and an overall mean for each participant. In addition, means were calculated for each of the three online cafés for the entirety of the participants. The affinity for technology varied between the participants. The main findings of the short questionnaire show that the participants with the highest affinity for technology were represented in the World Café “educational institution” and “community” (overall mean value 3.76). The World Café “leisure club” participants had a somewhat lower affinity for technology (overall mean value 3.65). The World Café “educational institution” and “community” had the lowest proportion of “low–tech participants”, with one participant each.

**Figure A2:**
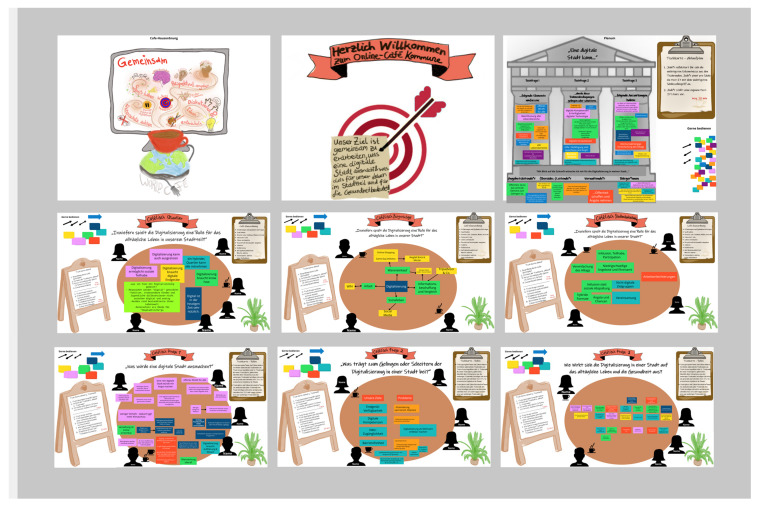
Labelled digital pinboard after the online World Café “community”.

Digital pinboard and memory protocols: For the analysis of the digital pinboard and memory protocols, all personal data (e.g., names of participants or institution names) on the respective digital pinboard were first anonymised, and a screenshot was taken. An example of the labelled digital pinboard of the World Café “community” is shown in the Appendix A (cf. Figure A2). For the analysis, the Post–its of the digital pinboard and memory protocols were imported into a MAXQDA project and subsequently evaluated by content analysis computer–assisted and theory–guided, according to Kuckartz [23]. With the help of the analysis software, the material was coded according to the categories and clustered. During clustering, the deductively formed categories were checked, adjusted, and supplemented with new categories (inductive categories) so that a final category system was formed as an analysis tool developing of the overarching definition of digital settings.

## 4. Discussion

Regarding the online-based realisation of the World Café method, advantages can be identified compared to an analogue hosting of the method, but also challenges, which will be discussed in the following regarding corresponding solutions. Further, benefits, challenges, and recommendations are derived and reflected below.

*Changes in methodology*: the methodological adaptation for the second and third online World Café can be perceived as positive and conducive to discussion due to the extended discussion times. As the participants could no longer discuss all table questions, but only two of three table questions, this is a deviation from the original method, according to Brown and Isaacs [13]. By presenting the results of the table questions and working together in plenary on all three table questions, the participants could still comment on the content of all questions and discussion items.

*Recruitment and target group:* Online formats can enable equal participation for different population groups through more low–threshold access to citizens’ dialogues. Nonetheless, it is also clear that despite lowered inhibition thresholds for participation via online formats, not all population groups can participate equally. Recruiting people with low socio–economic status is made more difficult when conducting online formats, although it should be emphasised that both analogue and online formats are challenging in this respect [24]. The recruitment of the conducted online World Cafés also faced challenges regarding particularly vulnerable target groups, e.g., in the settings of inclusion assistance and family counselling. For example, people who were accommodated in assisted living facilities could not be recruited. Due to a lack of time and resources associated with client care, participation in the online World Cafés was impossible. Recruiting several people from one facility to participate in an online World Café in parallel also made recruitment difficult. In addition, the recruitment of certain vulnerable target groups (e.g., homeless people) was ruled out due to the high requirements of online hostings, such as the availability of internet access and digital devices. Before deciding on a digital or analogue hosting of the World Café, the target group must be clearly defined and analysed to find a suitable hosting strategy.

A particular challenge was the World Café “Leisure Club” recruitment. Only two settings participated there, namely two different types of leisure clubs. The youth club setting could not be represented due to a lack of registrations. The preparation and realisation phases were in the summer or holiday period. Thus, many youth clubs could not participate because summer games or youth camps took place simultaneously. For this reason, the recruitment of participants from the sports club was extended to attract younger people as participants. In this way, heterogeneity of participants could be ensured. 

Compared to analogue hosting, recruitment can be seen as more flexible and expanded, as participation in the online World Café is possible regardless of location. This enables nationwide recruitment beyond regional structures. Especially for the target group envisaged in this research project, the expansion of recruitment was advantageous, as not only regional settings but also institutions located further away could be requested. Another advantage is that the online hosting did not require additional time (e.g., due to longer journeys).

A challenge in recruitment is the chosen timeframe for hosting the online World Cafés. This is also evident in the varying numbers of participants in the three online World Cafés. Due to the heterogeneous group constellation, consisting of different status groups from different institutions, the possible/optimal time windows for participation differ. Thus, August was deliberately chosen to enable the participation of teachers, pupils, and school administrators in educational institutions, as this is the holiday season. Concerning the leisure club setting, nonetheless, this means that leisure trips or summer camps were planned and carried out, e.g., in youth clubs, so participation through such an institution was not possible.

With the increased requirements of online hostings, such as the availability of internet access and digital devices as well as corresponding digital skills, there is also the danger of a recruitment bias. This could lead to recruiting more tech–savvy participants and excluding less tech–savvy setting members from participating in the event. The TAEG questionnaire was used to inquire about technology affinity to assess whether more tech–savvy participants were recruited. In general, it is advisable to obtain such an assessment via a short questionnaire in future research. To compensate for a more technology–savvy group of participants, the World Café can either be extended to a hybrid format or several events can be held in purely digital and analogue forms. The latter also makes it possible to examine commonalities and differences between “conversations that matter” in analogue and digital formats.

*Commitment and flexible planning*: Another aspect that seems interesting with online formats is the obligation to participate in the event. This refers both to showing up for the event and to active participation during the event. In this regard, especially regarding the online World Café for the setting of educational institutions, the cancellations at short notice, as well as the non–appearance of participants, should be emphasised. Here it would be interesting to look at analogue and digital formats to see whether and to what extent the number of registered but non–appearing participants differs. Due to higher cancellations of participants in digital formats, it would be of particular importance firstly to recruit more participants than the target number, and secondly to create a flexible planning structure that also contributes to the event’s success in the case of fluctuating numbers of participants.

From the point of view of the obligation to participate in the event, it is particularly noteworthy that the participants who come from a common institution were all present. The participants who did not show up or cancelled at short notice were only individual representatives of their institution.

*Time management and wording*: When preparing the timetable, it should also be considered that the group size influences the discussion time. The more people participate in the group discussion, the less time each person has to speak. In the case of more complex questions and the resulting need for discussion, there must be sufficient time to answer the questions. Thus, in the first online World Café “educational institution”, an initial time estimate was obtained; bottlenecks were identified and adjusted in the hosting of the other online World Cafés.

*Technical problems*: Another challenge in implementing an online World Café is the target group’s varying digital competencies. Due to the heterogeneity of the small groups, the level of digital competencies was heterogenous, too, so the successful use of technology varied and thus influenced active participation in the discussion. For example, several participants had problems opening Conceptboard via a tablet. As a solution, the table observer shared their screen at Zoom so that the participant could see the Conceptboard and, if needed, write down the participants’ verbally communicated discussion contributions on the Post–Its. This contributed to the fact that the participants could see the Conceptboard on their own and helped to ensure that they could participate to the same extent as the other participants. To address the different levels of digital competencies that were already suspected before the event, individual appointments were offered for Zoom training by the researchers. This was to enable prior exposure to possibly new technology. However, this offer was not taken up by any of the participants.

Another participant had difficulty creating Post-its on the digital pinboard even after the Conceptboard exercise. One person (in this case a senior citizen) needed technical support to actively participate in labelling the digital pinboard and received help from another setting member by participating from the same digital device. In principle, checking the technical implementation on different digital end devices is recommended in advance. If the selected tools do not work as planned on all digital end devices (e.g., Conceptboard a tablet), this should be pointed out in the invitation letter.

In addition, there were technical disruptions due to internet problems among the participants. This became apparent through faltering images or distorted voice recordings. This challenge cannot be solved entirely, even with the advice to ensure a stable internet connection, as related infrastructural challenges are not always the participant’s responsibility. To deal with any technical problems that may arise during the event, two different types of technical support should be offered during the online World Cafés. On the one hand, via the same communication medium (e.g., Zoom), on the other hand, via another internet-independent medium, such as the telephone, as a contact point for internet problems.

Overall, the technology problems arose independently of the recorded technology affinity, so a prediction of technology problems based on technology affinity could not have been made.

*Connection of perspectives and intergenerational interaction*: Regarding the goal of networking knowledge, the digital hosting of the World Café can be considered successful. The course of the online World Cafés shows that the linking of perspectives of the heterogeneous group of participants was done constructively. In particular, the respectful interaction between the generations should be emphasised here, which the overall café hosts and table observers found precious (e.g., mutual verbal assistant in setting Post-its or changing the Zoom window).

At this point, nonetheless, it should also be mentioned that the acquaintance among some café guests from the same institution fostered a familiar atmosphere that may have positively influenced the flow of discussion.

*Comparison with other studies with an online hosting of World Cafés*: The presented methodological findings align with other projects that have digitally implemented creative methods based on the World Café method [18,19]. Banfield et al. [19] also describe problems in recruiting participants for a digital realisation of 2,5 h World Cafés via Zoom, which underlines the relevance of recruiting café guests beyond the desired number of participants. Furthermore, Banfield et al. [19] express similar concerns regarding the possible exclusion of socially disadvantaged groups of people when implementing the World Café method online. In contrast to our World Cafés, the World Cafés conducted by Banfield et al. [19] did not help to build consensus among participants regarding the underlying research question because too many different aspects were discussed. In our case, this was not a significant problem, and especially the final plenary session was beneficial in ensuring a standard answer to the research question.

The one-hour World Cafés via Zoom conducted as part of the study by McKimm et al. [18] did not report any problems in recruiting participants but, on the contrary, rather too large groups of participants. However, it must be emphasized here that this is an international study. Nonetheless, McKimm et al. [18], like us, experienced technological difficulties in conducting their virtual World Cafés and reported the relevance of being able to adapt quickly and flexibly to such situations as an organisational team.

Laine-Gossin et al. [20] conducted a 90-min virtual World Café via Zoom based on four table rounds and visualised via virtual whiteboards. The authors do not mention any recruitment challenges. However, when conducting the event, they emphasise that the confidential space created by small groups becomes rather impersonal due to technical problems and limitations in the virtual environment, e.g., participants occasionally hesitate to speak because they feel inexperienced [20]. We did not have this experience in our online World Cafés. On the contrary, participants helped each other when technical problems arose and increasingly involved the people with technical problems, e.g., via the Zoom chat function. It should be noted that the mutually supportive participants were mostly from the same institutions and that support was probably favoured by sufficient discussion time. Similarly, Laine-Gossin et al. [20] emphasise the importance of making it clear that everyone’s contribution is valued for encouraging discussion, and this was also our experience in our online World Cafés. 

*Limitations of hosting online of World Cafés and potential future developments*: Hosting online World Cafés must be reflected regarding limiting factors. On the one hand, it was impossible to represent other groups of people who tend to be vulnerable in the online World Cafés due to recruitment challenges. The active participation and the documentation of the results on the digital pinboard during the World Cafés strongly depend on the respective competencies of the participants. Participants who are not proficient or confident in using Zoom or Conceptboard may contribute less content. In addition, technical malfunctions during the online World Café determine the discussion’s participation and flow. However, technical disruptions are often dependent on the participants’ infrastructure, which limits the support possibilities of the overall café hosts. In the future, implementing online World Cafés must be adapted to the target group and their competencies, and, if necessary, training opportunities must be integrated.

## 5. Conclusions

Hosting an online World Café was feasible and valuable for involving citizens in the definition of digital settings in the context of health promotions and prevention. However, specific challenges occur regarding equity of participation. The decision for or against hosting an online World Café and developing a recruitment strategy should consider the target group analysis. Overall, online World Cafés are suitable for involving different setting members in the discussion about digital transformation in different settings and for gaining knowledge about settings in the context of health promotion and prevention. Our results show that the following aspects have to be considered during the process of hosting online World Cafés:

- During the recruitment process, it must be ensured that future participants can access the internet and use digital devices. In addition, it should be ensured that appropriate digital skills are available to guarantee successful use of the tools. If this cannot be guaranteed, analogue or hybrid event formats should be considered.
- Support should be implemented to avoid excluding people without access to the internet or digital devices and people with low digital skills from the participatory research process. Thus, training opportunities or hybrid formats could be offered. Several events with purely analogue formats and purely digital formats are also conceivable.
- The schedule of an online World Café must be designed flexibly to react to spontaneous circumstances that may occur in digital formats compared to analogue formats. These circumstances could include fewer participants due to lower commitment or fluctuating numbers of participants due to possible technical disruptions.
- Considering the complexity of roles associated with digital hosting, it must be clearly defined which person takes on which role.

## Figures and Tables

**Table 1:** Overview of the person involved in the hosting team, their roles, and functions.

Person	Role	Function
A	Overall café host 1 (OCH 1) Table observer 1 (TO 1)	Holding the introductory presentation. Welcoming the participants. Moderating the contributions during the plenary discussion. Observing and taking protocol at one digital table.
B	Overall café host 2 (OCH 2) Table observer 2 (TO 2)	Welcoming the participants. Timekeeping and coordinating the contributions during the plenary discussion. Observing and taking protocol at one digital table.
C	Table observer 3 (TO 3)	Observing and taking protocol at one digital table and in the plenary session while OCH 1 and 2 moderate/coordinate.
D	Technical assistant (TA)	Being the contact person for technical difficulties. Realizing the technical management of the Zoom breakout sessions. Taking screenshots of the digital pinboards after each table round and plenary discussion.

**Table 2:** Examples of questions in the online World Café “educational institution”.

Phase	Question
Overarching question	What do we understand by a digital educational institution?
Café table 0 (institution-internal groups)	To what extent does digitalisation affect life in our educational institution?
Café table 1 (mixed groups)	What constitutes a digital educational institution from our common perspective?
Café table 2 (mixed groups)	From our common perspective, what contributes to the success or failure of digitalisation in educational institutions?
Café table 3 (mixed groups)	From our common perspective, how does digitalisation in educational institutions common teaching, learning and health?

**Table 3:** Characteristics of the participants in the online World Café.

Variable	Characteristics	Frequencies N (%)		
		*Educational Institution*	*Community*	*Leisure Club*
Age-group	20–29	6 (46.2)	3 (25.0)	7 (77.8)
	30–39	3 (23.1)	2 (16.7)	0 (0.0)
	40–49	2 (15.4)	3 (25.0)	0 (0.0)
	50–59	1 (7.7)	2 (16.7)	1 (11.1)
	60–69	0 (0.0)	2 (16.7)	1 (11.1)
	70–79	1 (7.7)	0 (0.0)	0 (0.0)
Sex	Male	6 (46.2)	8 (66.7)	3 (33.3)
	Female	7 (53.8)	4 (33.3)	6 (66.7)
	Diverse	0 (0.0)	0 (0.0)	0 (0.0)

## Data Availability

Not applicable.
